# Whole-Genome-Sequence-Based Evolutionary Analyses of HoBi-like Pestiviruses Reveal Insights into Their Origin and Evolutionary History

**DOI:** 10.3390/v15030733

**Published:** 2023-03-11

**Authors:** Semmannan Kalaiyarasu, Niranjan Mishra, Saravanan Subramaniam, Dashprakash Moorthy, Shashi Bhusan Sudhakar, Vijendra Pal Singh, Aniket Sanyal

**Affiliations:** 1 ICAR—National Institute of High Security Animal Diseases, Bhopal 462022, India; kalai_82vetmic@yahoo.co.in (S.K.); dr.dassprakash@gmail.com (D.M.); sudha.vet@gmail.com (S.B.S.); vijendra61@gmail.com (V.P.S.); aniket.sanyal@gmail.com (A.S.); 2 ICAR—National Institute on Foot-and-Mouth Disease, Bhubaneswar 752050, India; saranvirol@gmail.com

**Keywords:** HoBi-like pestivirus, HoBiPeV, evolutionary analysis, phylogeny, whole-genome sequence, selection pressure, recombination

## Abstract

HoBi-like pestivirus (HoBiPeV), classified under *Pestivirus H* species, is an emerging cattle pathogen of high economic impact. However, the origin and evolution of HoBiPeV are not very clear due to a lack of full genomic sequences from diverse clades. This study aimed to determine full-genome sequences of HoBiPeV strains of three novel clades (c, d and e) and perform full-genome-based genetic and evolutionary analyses. Bayesian phylogenetic analyses herein confirmed the existence and independent evolution of four main HoBiPeV clades (a, c, d and e) globally, with genetic divergence ranging from 13.0% to 18.2%. Our Bayesian molecular clock estimates revealed that HoBiPeV most likely originated in India, with a dated tMRCA of 1938 (1762–2000), evidencing a more recent origin of HoBiPeV. The evolution rate of HoBiPeV was estimated to be 2.133 × 10^−3^ subs/site/year at full-genome level but varied widely among individual genes. Selection pressure analyses identified most of the positively selected sites in *E2*. Additionally, 21.8% of the ORF codon sites were found under strong episodic diversifying selection, providing first evidence of negative selection in HoBiPeV evolution. No recombination event was evident for HoBiPeV-c, d and e strains. These findings provide new insights into HoBiPeV origin and evolutionary history for better understanding the epidemiology and host–pathogen interactions and stimulate vaccine research.

## 1. Introduction

Infection of cattle with bovine pestiviruses causes respiratory, reproductive and enteric diseases and inflicts significant economic losses in the cattle industry around the world. Belonging to the genus *Pestivirus* under family *Flaviviridae*, these viruses are classified into three pestivirus species, namely *Pestivirus A* (bovine viral diarrhoea virus 1, BVDV-1), *Pestivirus B* (bovine viral diarrhoea virus 2, BVDV-2) and *Pestivirus H* (HoBi-like pestivirus, HoBiPeV) [1]. Although less widespread globally than BVDV-1 and BVDV-2, HoBiPeV is currently prevalent in many countries. HoBiPeV genomic RNA is approximately 12.3 kb in length and contains a single large open reading frame (ORF) flanked by 5′- and 3′-untranslated regions (UTR). The ORF encodes a polyprotein of approximately 3900 amino acids that is processed into 12–13 proteins (*N^pro^*, *C* (capsid), envelope proteins *E^rns^*, *E1* and *E2* and non-structural proteins *p7*, *NS2*, *NS3*, (*NS2–3*), *NS4A*, *NS4B*, *NS5A* and *NS5B*) by viral and cellular proteases [2].

HoBiPeV was discovered in 2004 in Germany as a cell culture contaminant originating from Brazilian foetal bovine serum (FBS) and the strain was named HoBi_D32/00 [3]. Subsequently, HoBiPeV was detected in commercial FBS lots from Brazil, Argentina, Mexico, Canada and Australia [4,5]. Nevertheless, there is evidence that HoBiPeV was present in Brazilian buffalo much earlier in 1996 [6]. Thus far, natural infection of cattle with HoBiPeV has been detected in three continents: South America (Brazil and Argentina), Europe (Italy and Turkey) and Asia (Thailand, India, Bangladesh and China) [5,7,8,9,10,11,12,13,14]. Although HoBiPeV infection displays similar clinical signs to BVDV-1 and BVDV-2 infections, it has been associated with reproductive disease [15,16], severe respiratory disease [9,17], gastroenteritis and economic losses in cattle farms [14,18] and mucosal disease in cattle [19,20,21,22]. Recent reports of HoBiPeV-associated cattle deaths in China [14] and India [22] are a great concern for the global cattle industry as, currently, there is no commercially available vaccine against HoBiPeV. Aside from cattle, natural HoBiPeV infection has also been detected in buffalo [6,7], sheep and goats [23].

Originally, it was thought that HoBiPeVs were genetically less diverse. However, based on *5′-UTR* or *N^pro^* genomic sequence analysis, HoBiPeV strains have been classified into five distinct genetic lineages or clades (HoBiPeV-a to HoBiPeV-e) thus far [11,22,23,24,25,26]. The strains of the HoBiPeV-a clade have been widely detected in infected cattle in Brazil, Italy, Turkey, Thailand and China [4,8,12,13,27], while the strains of HoBiPeV-b clade originated in Bangladesh [10]. Surprisingly, the highly divergent HoBiPeV strains of the other three clades (c, d and e) have been detected in cattle exclusively in India [11,22].

Phylogenetic analysis of pestiviruses is vital for classification of novel and emerging viruses, tracing the origin of outbreaks and revealing their evolutionary history. However, little is known about the evolution of HoBiPeVs, and the evolutionary relationships among HoBiPeVs have not been unambiguously determined due to the lack of adequate full-genome sequence data from different clades with diverse geographical origin. Thus far, full genomic sequences are available only for the strains of HoBiPeV-a clade. Hence, the aim of this study was to determine and analyse the complete-genome sequences of the strains of HoBiPeV-c, HoBiPeV-d and HoBiPeV-e clades and decipher the evolutionary relationships among HoBiPeVs based on all available complete-genome sequences. We performed Bayesian evolutionary analysis, selection pressure analysis and recombination analysis to unravel the origin and evolution of HoBiPeVs. Based on Bayesian analysis of the time of most recent common ancestor (TMRCA), we show that HoBiPeV most likely originated in India, with the emergence dating to 1938 (1762–2000). Additionally, we show that HoBiPeVs most likely evolved independently into four clades, and >10.0% divergence at the full-genome level may be considered for classification of genetic clades of HoBiPeVs. Furthermore, we determined the effect of purifying selection, which was uniformly spread over all the genomic regions, in shaping HoBiPeV evolution. The findings here expand our knowledge about the origin and evolutionary history of HoBiPeV.

## 2. Materials and Methods

### 2.1. Virus Strains

The complete-genome sequences of the three novel HoBiPeV strains (IndBHA5309/12, IndABI15385/12 and Ind/TN-1214/19) originating from cattle in India were determined and analysed in this study. HoBiPeV IndBHA5309/12 is an archived strain isolated from clinical samples collected from cattle in western India in 2012 and grown in Madin–Darby bovine kidney (MDBK) cells [11]. HoBiPeV IndABI15385/12 strain was isolated from clinical samples from cattle in central India in 2012 and cultured in MDBK cells [11]. HoBiPeV Ind/TN-1214/19 strain was isolated from crossbred Jersey cattle showing mucosal-disease-like lesions in southern India in 2019 and cultured in MDBK cells [22]. These strains represented all three highly divergent HoBiPeV clades (HoBiPeV-c, d and e) reported from India [11,22].

### 2.2. Next-Generation Sequencing, De Novo Assembly and Genome Annotation

The RNA obtained from low-passage (3rd passage) HoBiPeV strains in MDBK cells were sequenced using an Illumina NextSeq 500 platform (Illumina, San Diego, CA, USA) through outsourcing (M/s Genotypic Technology Pvt. Ltd., Bengaluru, India and Eurofins genomics India, Bengaluru). RNA extraction was performed using the TruSeq RNA Sample Preparation Kit (Illumina). Library preparation was performed following the IlluminaTruSeq RNA library protocol outlined in “TruSeq RNA Sample Preparation Guide” (Illumina). Following second-strand cDNA synthesis, the cDNA was cleaned up using HighPrep PCR (MAGBIO) and Illumina adapters were ligated to the cDNA molecules after end repair and adenylation of the cDNA ends. The prepared library was quantified using Qubit and validated for quality by running an aliquot on High Sensitivity Bioanalyzer Chip (Agilent, Santa Clara, CA, USA). The Illumina paired end (2 × 150 bp) raw reads were quality-checked using FastQC [28]. Illumina raw reads were processed by an in-house script (SeqQC) for adapters and low-quality bases trimming towards 3’-end.

Illumina reads were assembled using SPAdes program [29], intended for de novo assembly after error-correction of sequenced reads. Contig generation was completed using Velvet [30], and virus-related contigs were separated from the assembly. Mapping of high-quality reads on reference genomes (FJ040215: Th_Khonkaen/2004, AB871953: D32_00/HoBi) was completed using Bowtie [31]. De novo assembled sequences were also analysed by comparison to reference HoBiPeV sequences from GenBank via the BLASTn and BLASTx tools of the National Center for Biotechnology Information (NCBI) website. Proteins were predicted from the assembled genome using Prodigal [32].

For confirmation of 5′ and 3′-UTR end sequences of HoBiPeV strains generated through Illumina ngs, we additionally employed RNA ligation method prior to RT-PCR, cloning and Sanger sequencing, as per the previously reported method [33]. Nucleotide sequencing (Sanger sequencing) of three independent clones of each DNA fragment was carried out in an automatic DNA sequencer (ABI 3130, Applied Biosystems, MA, USA) at ICAR-NIHSAD, Bhopal. The three complete-genome sequences of HoBiPeVs generated in this study have been deposited in GenBank under accession numbers OQ411019, OQ411020 and OQ411021.

### 2.3. Maximum Likelihood Analysis

The nucleotide sequences of complete genomes of 20 HoBi-like pestiviruses and 14 reference pestiviruses available from the GenBank database (Supplementary Table S1) and the three complete-genome sequences of HoBiPeVs generated in this study were aligned using the MUSCLE algorithm. The mean and pairwise divergence of the complete genome and various coding regions were then computed. To assess the evolutionary relationships among HoBi-like and other pestiviruses, phylogenetic trees were inferred by maximum likelihood (ML) method based on the nucleotide alignment of the full-length sequences using MEGA software v. 11 [34]. The ML phylogeny was produced under GTR evolution model, with rate variation following a gamma distribution, as determined by model finder, and robustness of tree topology was assessed by bootstrap analysis with 1000 iterations. The nearest-neighbour interchange (NNI) technique was used to conduct a heuristic tree search, and a neighbour-joining (NJ) tree was selected as the starting tree.

### 2.4. Recombination Analysis

The full-genome sequence alignment of HoBi-like pestiviruses was examined for any signs of recombination using RDP5 software v. 5.30 [35]. The analysis was carried out using various methods available and their default parameters. Recombination events were only deemed proven if they were identified by at least four of the seven available algorithms (RDP, Geneconv, BootScan, MaxChi, Chimaera, SiScan and 3Seq) with default values. The pairwise homoplasy test (PHI test) was additionally utilised to look for recombination event evidence [36]. This test calculates a *p*-value and evaluates the significance of evolutionary disagreement across locations in an alignment. A *p*-value of less than 0.01 indicates the presence of recombination evidence. With a low false-positive rate, the PHI test is particularly effective at determining whether recombination has occurred or not across a broad range of sequence divergence.

### 2.5. Selection Pressure Analysis

Three likelihood approaches were employed to determine positive selection pressure at certain codon sites: the single-likelihood ancestor counting (SLAC) method, the fixed effects likelihood (FEL) method and a Bayesian strategy called FUBAR. The ratio of non-synonymous (dN) to synonymous (dS) substitutions per site (ratio: dN/dS) was used to calculate the strength of selection pressure. In general, posterior probability >0.9 for FUBAR and *p* < 0.1 for SLAC strongly imply positive selection. The mixed effects model of evolution (MEME) was used to identify the codon sites that were the subject of episodic diversifying selection. Strong evidence of selection was accepted at significance levels (*p* < 0.05). All the analyses were carried out using the online Datamonkey webserver [37].

### 2.6. Bayesian Analysis

The reconstructed ML nucleotide trees were utilised in TempEst [38] to produce linear regression plots of the years of sampling versus root-to-tip distance in order to examine the temporal signal. Using time-stamped sequence data with a relaxed and uncorrelated lognormal clock under the Bayesian Markov chain Monte Carlo (MCMC) method, BEAST version 1.10.4 [39] was used to estimate the rates of evolutionary change (nucleotide substitutions per site per year) and times of circulation of the MRCA (years). The GTR + G + I nucleotide substitution model and an exponential coalescent population with default priors were used. The exponential prior offered a limited HPD range and proper parameter convergence among the many coalescent models examined. For ORF and whole genomes, three independent runs of 200 million generations were carried out, their convergence was evaluated and the log and tree files were then combined with the aid of LogCombiner. After a single run of 200 million generations, convergence was observed for specific coding regions. An asymmetrical-state transition model was used to predict discrete-state ancestral reconstruction of viral sampling locations and migration rates between geographic regions [40]. Using TreeAnnotator 1.7.3, the MCMC chains were condensed to reconstruct the MCC trees. FigTree application v.1.4.0 was used to visualise and colour trees. The statistical uncertainty in the parameter estimates across the sampled trees was reflected in the 95% highest probability density (HPD) intervals.

## 3. Results

### 3.1. HoBiPeV Genome Characteristics

The complete-genome sequences of three HoBiPeV strains from India were determined in this study and the genomes displayed a classic pestivirus genome organization, comprising one open reading frame (ORF) flanked by untranslated regions (UTRs) at either end, the 5′-UTR and 3′-UTR (Table 1). The lengths of the three complete genomes of Indian HoBiPeVs were determined to be 12,372, 12,251 and 12,259 nt, respectively, for strains of HoBiPeV-c (IndABI15385/2012), HoBiPeV-d (IndBHA5309/2012) and HoBiPeV-e (HoBiPeV Ind/TN-1214/19). The ORF length of all three HoBiPeVs was alike and comprised 11,700 nt, which encoded a polyprotein encompassing 3899 aa. No viral gene duplications or cellular sequence insertions were found in the genomic RNA of these three HoBiPeVs.

The *5′-UTR* of IndABI15385/12, IndBHA5309/12 and HoBiPeV Ind/TN-1214/19 consisted of 393 nt, 373 nt and 367 nt, respectively (Table 1). It is noteworthy that the 393 nt length of *5′-UTR* of IndABI15385/12 is the highest among the HoBiPeV strains reported so far, which is 2 nt longer than Chinese strain HN1507. The *3′-UTR* of IndABI15385/12 consisted of 279 nucleotides with AT content of 68.1%, while *3′-UTR* of IndBHA5309/12 consisted of 178 nucleotides with AT content of 64.61% and Ind/TN-1214/19 consisted of 189 nucleotides with AT content of 63.5%. As the length of *3′-UTR* has been reported to be variable between 134 and 255 nts for HoBiPeV strains, the 279 nt length of *3′-UTR* in IndABI15385/12 found here is the longest among the HoBiPeV strains and other bovine pestiviruses (BVDV-1 and BVDV-2) reported so far and is 15 nt longer than HoBiPeV strain Th/04_KhonKaen (Table 1).

The complete-genome nucleotide sequence identity between IndABI15385/12 (HoBiPeV-c) and IndBHA5309/12 (HoBiPeV-d) was found to be 79.8%, whereas it was 85.1% between IndABI15385/12 (HoBiPeV-c) and Ind/TN-1214/19 (HoBiPeV-e) and 82.3% between IndBHA5309/12 (HoBiPeV-d) and Ind/TN-1214/19 (HoBiPeV-e) (Supplementary Table S2). The amino acid identity at the complete ORF level was 84.5% between IndABI15385/12 (HoBiPeV-c) and IndBHA5309/12 (HoBiPeV-d), whereas it was 89.9% between IndBHA5309/12 (HoBiPeV-d) and Ind/TN-1214/19 (HoBiPeV-e) and 91.3% between IndABI15385/12 (HoBiPeV-c) and Ind/TN-1214/19 (HoBiPeV-e). The nucleotide and amino acid sequence identity between these strains and strains of the HoBiPeV-a clade are shown in Supplementary Table S2. The amino acid mutations unique to the strains of HoBiPeV-c, d and e clades analysed here are shown in Table 2.

### 3.2. Phylogenetic Relationships

The complete-genome sequences of three HoBiPeV strains collected from India and determined in this study were aligned with 20 full-genome sequences of HoBi-like viruses and 14 representative strains from other pestivirus species available in GenBank. The analysed HoBiPeV strains were collected between 2000 and 2019 (Table 1). As per the history available, the strains are from six countries or regions, including Brazil (*n* = 10), China (*n* = 2), Thailand (*n* = 1), Italy (*n* = 6), India (*n* = 3) and South America (*n* = 1).

**Figure 1:**
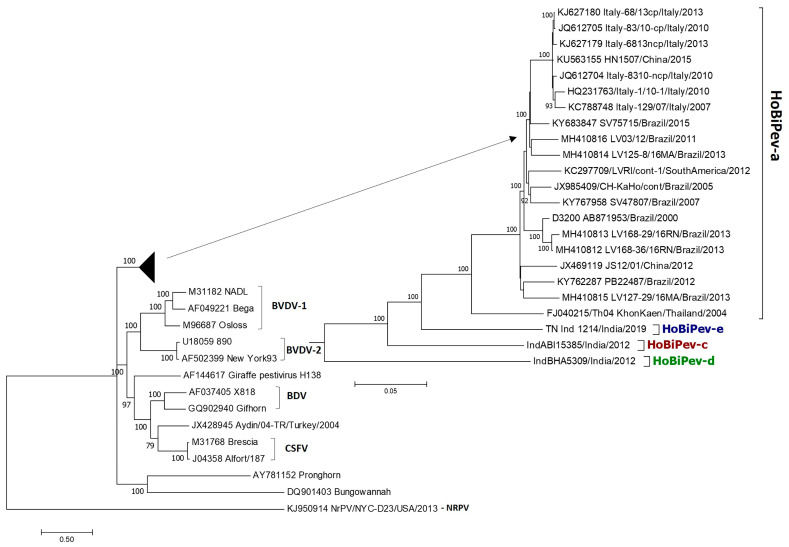
Phylogenetic tree based on full-genome sequences of HoBiPeV genetic lineages a, c, d and e depicting relationships among HoBiPeVs and between the HoBiPeV and other pestiviruses. Tree topology was assessed by bootstrap analyses with 1000 replicates, and values are indicated in nodes. The sequences of HoBiPeV clades c, d and e were determined in this study and other sequences are from GenBank (Table 1). The scale bar indicates 5% of nucleotide divergence.

The maximum likelihood (ML) phylogenetic tree (Figure 1) based on whole-genome sequence analyses showed that globally circulating HoBiPeVs can be resolved clearly into four clades (a, c, d and e) and supported by strong bootstrap values (100%). Since the entire genome of the HoBiPeV-b clade is not currently available, it was excluded from the analysis. In the ML tree, the strain of the HoBiPeV-d clade formed an ancestral node and showed percent nt divergence of 17.9–18.7, 18.2–17.7 and 18.2–17.7, respectively, from clades HoBiPeV-a, HoBiPeV-c and HoBiPeV-d, which is the highest inter-clade diversity observed. Strains HoBiPeV-e and HoBiPeV-c showed 12.7–13.4% and 14.3–15.0% divergence, respectively, from strains of clade HoBiPeV-a. Strains HoBiPeV-e and HoBiPeV-c had a nt divergence of 14.8%. The average genetic divergence among HoBiPeV-a clade was found to be 4.0%. Minimum inter-clade mean genetic distance was observed between HoBiPeV-a and HoBiPeV-e (13.0%), and maximum mean genetic distance was observed between HoBiPeV-a and HoBiPeV-d (18.2%). Based on our results, a cut-off of >10.0% divergence at the full-genome level may be considered for classification of genetic clades of HoBi-like pestivirus. For the HoBiPeV genome dataset, percent nt and aa divergence at various coding regions were determined and shown in Table 3. As anticipated, the *E2* region had the highest mean nt and aa diversity, followed by the *NS5A* coding region. With the exception of *p*7, there was a high degree of phylogenetic congruence between the whole-genome-based ML tree and those built using various gene sections.

### 3.3. Selection Pressure

We simultaneously used three techniques (SLAC, FEL and FUBAR) to analyse the selection pressure acting on the codon sites of the ORF of HoBi-like pestivirus. In general, the calculated dN/dS ratios for the ORF (0.126) and several genes (range from 0.060 to 0.230) of HoBiPeVs were all less than 1.0, indicating modest selection pressure (Table 4). Among the different genomic regions, a higher dN/dS ratio was observed for *E2* and the lowest for *C*. Four codon positions (N^pro^-114, E2-20, NS5A-304 and NS5B-203) were found to be under positive selection by both SLAC and FUBAR. In addition, positions E2-89 and NS5B-179 were identified by FUBAR and SLAC, respectively. FEL identified 19 sites to be under selection pressure, most of which (11 out of 19) are located within the *E2* protein.

Additionally, the MEME likelihood approach was used to identify sites under episodic diversifying selection. The approach projected episodic pressure at 68 codon sites in HobiPeVs. *E2* (n = 15) had the second-highest number of codon sites subject to episodic selection, followed by *NS2/3* (n = 20), and 21.8% of codon sites in the ORF were found to be under strong purifying selection, which indicated a major role for negative selection in shaping their evolution. Negatively selected sites were identified using the SLAC method. Further, negative Tajima’s *D* values (−1.181018) and low nucleotide diversity (0.070774) among the whole-genome sequences of HobiPeV strains point to low frequency of nucleotide polymorphism and strong selection and/or population size expansion. With the exception of *E^rns^*, which had the lowest value, our analysis demonstrated that the effect of negative selection was nearly uniformly spread over various genomic regions.

### 3.4. Evidence of Recombination

We investigated possible recombination events within Indian HoBiPeV genomes and other reference genomes using RDP5 software. The results showed that five recombinant events resulted in emergence of four recombinant viruses, which were detected in the current study with a *p*-value of 0.01 and a recombinant score of >0.4 (Table 5). HoBiPeV strain Italy-1/10 (HQ231763) identified in Italy was found to be a double recombinant. In most of the cases, it is also possible that the major parent could be the actual recombinant. The recombination sites were distributed non-randomly along the genome and the recombination breakpoints were detected in *5′-UTR*, *E2*, *NS2/3*, *NS4B*, *NS5A* and *3′-UTR*. All the recombination events were found only among the strains of HoBiPeV-a clade, and none of the Indian HoBiPeVs (HoBiPeV clade-c, d and e) sequenced in this study showed recombination. The PHI test, on the other hand, did not find statistically significant evidence for recombination (*p* = 0.09). Nearly the same values were obtained when we performed the evolutionary analysis after excluding putative recombinant sequences, suggesting that the recombinant fragment was shorter and had little effect on the estimate.

### 3.5. Temporal and Spatial Structure

**Figure 2:**
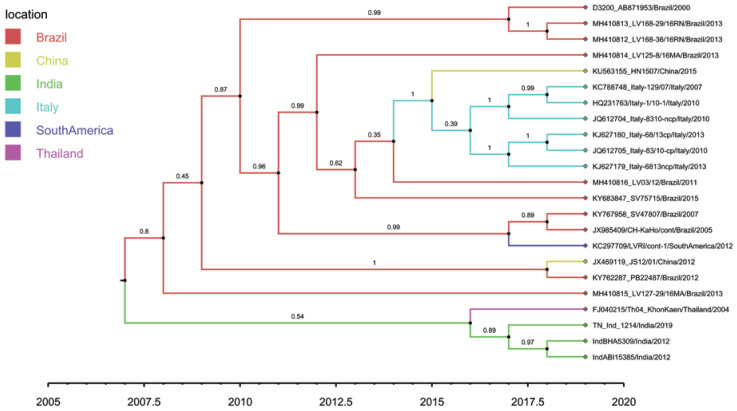
Tip-labelled maximum clade credibility phylogenetic tree reconstructed from 23 globally distributed HoBiPeV full genomes, including sequences from the three divergent clades (c, d and e) determined in this study. The rates of HoBiPeV evolutionary change (nucleotide substitutions per site per year) and times of circulation of the MRCA (years) were estimated using time-stamped sequence data with a relaxed and uncorrelated lognormal clock under the Bayesian Markov chain Monte Carlo (MCMC) method, BEAST version 1.10.4 [39]. The branches are coloured based on the most probable location of the descendent nodes. The sequences from this study are highlighted with green colour.

Root-to-tip regression analysis of the genetic distances of HoBi-like pestiviruses against sampling time, conducted using TempEst software, produced a correlation coefficient range of 0.31 to 0.45 and coefficient of determination (R2) of 0.09 to 0.20 for the whole genome, the ORF and different coding regions (Table 6). The 5′-UTR region showed the maximum temporal signal, followed by *E1*, *C*, *NS2/3*, *N^pro^* and the whole genome. Overall, although not very strong, a positive correlation was observed, thus suggesting a significant relationship between genetic divergence and time. A whole-genome-based maximum clade credibility (MCC) tree reconstructed in this study revealed two main clusters: one contains all the strains of HoBiPeV-a clade except strain Th04_KhonKaen, which, in turn, was placed in a separate cluster along with the strains of HoBiPeV-c, d and e (Figure 2). The *E2* gene-derived MCC tree, on the other hand, revealed the same topology as the ML tree.

The relative nucleotide substitution rates at all three codon positions in ORF showed that substitutions were more frequent at the third codon position (2.242, 95% HPD 2.201–2.286) compared to the first (0.499, 95% HPD 0.461–0.535) and second (0.259, 95% HPD 0.233–0.285), as expected. The mean rate of evolutionary change in the HoBiPeV was estimated to be 2.133 × 10^−3^ subs/site/year (95% HPDs of 2.181 × 10^−4^ 3.933 × 10^−3^ subs/site/year) for the full genome and 2.074 × 10^−3^ subs/site/year (95% HPDs of 2.833 × 10^−4^ 3.759 × 10^−3^ subs/site/year) for the ORF (Table 6). The substitution rate of each coding region of HoBiPeV was compared and the estimates varied in the following order: *E2*, *E^rns^*, *NS5B*, *NS4A*, *N^pro^*, *NS4B*, *NS2/3*, *NS5A*, *E1*, *C* and *P7*. Surprisingly, the *E2* coding region had a nucleotide substitution rate considerably lower than other coding regions and the whole genome. The *P7* protein coding region was found to have the highest evolutionary rate, and, among the structural coding regions, the *C* region showed the fastest evolutionary rate followed by the *E1* region.

The results of our Bayesian phylogenetic analysis based on whole-genome sequences (Table 6) suggested that HoBi-like viruses most likely emerged in India (root state posterior probability (RSPP) = 42.33%), with a recent common ancestor in 1938 CE (95% credibility interval: 1762–2000 CE). When ORF alone was used in the analysis, HoBiPeVs from India received an RSPP of 40.86% and HoBiPeVs found in Brazil received 39.69%, with a tMRCA of 1942 CE (95% credibility interval, 1800–1998 CE). Similar to this, the estimated tMRCA derived from individual gene sequences covered the period from 1962 to 1989, with the exception of the *N^pro^* and *E2* genes, which yielded estimates from much earlier periods. In our analysis, the tMRCA estimate for the *E2* gene dates to the 12th century and that for the *N^pro^* region to the 18th century.

## 4. Discussion

HoBi-like pestiviruses are a serious emerging threat to cattle populations, but only a few full-genome-sequence-based studies are available. Thus far, HoBiPeV strains have been classified into five clades or genotypes (a–e) based on partial *5′-UTR* or *N^pro^* sequences, but full genomic sequences are available only for strains of the HoBiPeV-a clade. This work was conducted to genetically characterise the representative strains from HoBiPeV clades c, d and e from India at the entire-genome level and determine the evolutionary relationships of HoBiPeVs because strains of three out of the five lineages described thus far have been circulating in India. Here, we report the first complete-genome sequences of strains belonging to the HoBiPeV-c, HoBiPeV-d and HoBiPeV-e clades and the full-genome-based evolutionary analyses of HoBiPeVs.

Use of at least two-genetic-regions-based phylogenies is advised for classification of HoBiPeV lineages, and trees-based datasets combining the *5′-UTR* and *N^pro^* genetic regions or 5′-UTR-N^pro^-E2 provide a robust phylogeny with higher statistical support [11,24]. The previously described HoBiPeVs from Brazil, Italy and Thailand were found to be very closely related and have formed a single genetic lineage, known as the HoBiPeV-a clade [24,26]. Subsequently, the HoBiPeV strains from cattle in Bangladesh were assigned to the HoBiPeV-b clade [10]. The strains of two highly divergent and novel linages from India were then described in 2014 and named the HoBiPeV-c and -d clades based on concatenated datasets of the *5′-UTR* and *N^pro^* sequences [11]. Recently, it has been found that India is home to strains of yet another unique lineage, the HoBiPeV-e clade [22]. However, evolutionary analyses between bovine pestiviruses and among HoBiPeVs have not been deciphered clearly as different phylogenetic relationships are inferred from analysis of different genetic regions [41] and for HoBiPeV. Thus far, only a few studies are available based on complete-genome-based phylogenetic analysis of strains belonging to only the HoBiPeV-a clade [26,42].

In the present study, we showed that the HoBiPeV clade formed a sister-clade with two other bovine pestivirus species, BVDV-1 and BVDV-2, based on phylogenetic analysis of full-length pestivirus genomic sequences, including the complete genomic sequences of four HoBiPeV clades, confirming the earlier hypothesis that these bovine pestiviruses have emerged from a common ancestor [24,41,42]. Furthermore, based on whole-genome sequence analyses, this study confirms the existence of four HoBiPeV main clades (a, c, d and e) globally, with the HoBiPeV-d strain found in India forming an ancestral node. The average genetic divergence among the strains of the HoBiPeV-a clade was found to be 4.0%, which is inconsistent with a previous study [26]. However, our results here showed the minimum inter-clade mean genetic divergence between HoBiPeV-a and HoBiPeV-e (13.0%) and the maximum mean genetic divergence between HoBiPeV-a and HoBiPeV-d (18.2%), suggesting that a cut-off of >10.0% divergence at the full-genome level may be considered for classification of genetic clades of HoBi-like pestiviruses.

The origin and evolutionary divergence of HoBiPeV have been a matter of debate recently. In this study, we performed a Bayesian molecular clock estimate based on full-genome sequences from four HoBiPeV main clades, showing that HoBiPeV most likely originated in India with a tMRCA of 1938 CE (95% credibility interval: 1762–2000 CE) approximately. Additionally, based on ORF analysis, HoBiPeVs revealed a tMRCA of 1942 CE (95% credibility interval, 1800–1998 CE), while the estimated tMRCA derived from individual gene sequences covered the period from 1962 to 1989, with the exception of the *N^pro^* (18th century) and *E2* (12th century). The studies on evolutionary analyses with regard to the origin and dating of tMRCA of HoBiPeVs have so far been based on either single-gene, *5′-UTR* [43], *N^pro^* [26] or combined datasets of 5′-UTR-N^pro^-E2 [24] wherein various estimates of tMRCA were reported. For instance, a previous study [24] reported a tMRCA of 1880 (HPDs, 1651–1993) based on combined datasets of 5′-UTR, N^pro^ and E2 gene. In contrast, another study [26] reported a tMRCA of 1566 (95% HPD 1001–1901) based on the *N^pro^* gene, while a recent study [43] reported a tMRCA of 1952 (95% HPD 1905–1985) based on the *5′-UTR*.

The dated tMRCAs of earliest origin, 1566 or 1880, suggested in previous studies [24,26], are much earlier than the date (1938) we estimated in this study. Meanwhile, the origin date of 1952 speculated by a recent study [43] is closest to the date of origin estimated in our study. This could be because previous Bayesian analyses used mostly HoBiPeV-a sequences or included some available *5′-UTR*/*N^pro^* sequences from clades c and d [24,26,43]. In contrast, this study is based on Bayesian analysis of full-genome sequences of the HoBiPeV strains of all four main clades (a, c, d and e). In addition, our analyses show that the genomic regions chosen for estimating age of tMRCA, along with quantity of sequences and time period, have a significant influence on the estimates. It is important to keep in mind that the majority of reported dates of tMRCA, including those reported in this study, are linked to broad HPDs; hence, it is likely that the mean tMRCA date does not accurately represent the actual date. Nevertheless, our findings provide evidence of a more recent origin of HoBiPeV, which has evolved independently into four clades and adds credence to the earlier notion that HoBiPeV most likely originated in India before its emergence in other parts of the world [22,26,43]. A limitation of this study is the sparse evolutionary comparison of sequences at the full-genome level due to their unavailability in public databases, and, hence, more complete-genome-based analyses will produce more reliable and accurate estimates and greater evolutionary knowledge in the future.

Although several hypotheses have been made, the dispersal of HoBiPeV following its origin is not very clear so far. The association of HoBiPeV lineages with geographic regions found earlier and in this study suggests that HoBiPeVs were circulating in cattle populations long before first being discovered [11,26]. Previous studies hypothesized that HoBiPeV evolved independently in the Indian subcontinent and elsewhere [11,25] or was introduced into Brazil coinciding with intensive importation of water buffalo and indicine cattle (*Bos indicus*) from Asia in the 20th century [26]. A recent study based on Bayesian analysis speculated that Italy is the hub of spread of HoBiPeV as it is the junction between Europe and Asia for export and import [43]. However, due to the range of genetic divergence found at the full-genome level between the HoBiPeV strains (clades c, d and e) circulating in India and in other parts of the world (clade a) in this study and the lack of full-proof epidemiological links, we suppose that, although HoBiPeV most likely originated in India and evolved independently, its spread to different geographical regions remains inconclusive. Indeed, India has a long trade history of cattle germplasm imports from Brazil and several European countries for upgrading the native cattle breeds (*Bos indicus*). Moreover, despite the high prevalence of HoBiPeV in Brazil, only strains of the HoBiPeV-a clade with little genetic variation have been identified to date [44].

Assessment of the rates of evolution of HoBiPeVs in this study revealed that the mean nucleotide substitution rate of HoBiPeVs is 2.133 × 10^−3^ subs/site/year (95% HPDs of 2.181 × 10^−4^ 3.933 × 10^−3^ subs/site/year) for the full genome and 2.074 × 10^−3^ subs/site/year (95% HPDs of 2.833 × 10^−4^ 3.759 × 10^−3^ subs/site/year) for the ORF, while the rates varied widely at the individual-gene level. Although the evolution rate for HoBiPeV at the full-genome level is not available so far for comparison, the rate calculated in our study approximates with the evolutionary rate (1.4 × 10^−3^ subs/site/year) reported for BVDV-1 and BVDV-2 at the full-genome level [45]. In contrast, a relatively higher rate of evolution (approximately 1.1 × 10^−2^ subs/site/ year) for HoBiPeV has been reported in a previous study [43], which may be because the previous study was based on evolutionary analysis of *5′-UTR* sequences. Interestingly, based on individual gene analysis, we found that gene-coding *P7* protein has the highest evolutionary rate, and, among the genes of structural coding regions, the *C* gene showed the fastest evolutionary rate and gene-coding immunodominant *E2* protein showed the lowest evolution rate among the coding region genes. The observed low evolutionary rate of *E2* is perplexing as it is the most variable gene compared to other coding region genes.

Another focus of our study was selection pressure analysis of HoBiPeV ORF, including the envelope glycoprotein *E2* gene, which is immunodominant and generates virus-neutralizing antibodies in the host. We observed modest selection pressure for HoBiPeV as the calculated dN/dS ratios for the ORF (0.126) and several genes (ranging from 0.060 to 0.230) of HoBiPeVs were all <1.0. However, among the different genomic regions, a higher dN/dS ratio was observed for *E2*, and most of the sites under positive selection pressure (11 of 19) are located within the *E2* protein. In a previous study also, HoBiPeV *E2* was reported to have the highest number of sites (11 of 18) under positive selection pressure [26]. Our findings also agree with the results of previous studies on two other related bovine pestiviruses, BVDV-1 and BVDV-2 [46,47]. Additionally, we conducted episodic selection pressure analysis of HoBiPeV as, in addition to positive selection, many codon sites may also experience selection in a restricted number of branches, designated as episodic diversifying selection, associated with selection pressure at the host–pathogen interface [48]. Our results showed episodic pressure at 68 codon sites in HoBiPeVs, of which *NS2/3* (n = 20) had the highest and *E2* (n = 15) had the second-highest number of codon sites subject to episodic selection. Interestingly, 21.8% of the codon sites in the ORF were found to be under strong purifying selection, suggesting a major role for negative selection in shaping HoBiPeV evolution. Episodic diversifying selection has been shown to play a major role in shaping evolution of foot and mouth disease virus (FMDV) VP1 and VP3 [49]. The present study reports a role of episodic diversifying selection in HoBiPeV evolution for the first time. However, identifying the importance of this selection requires further research.

Genetic recombination is an important source of genetic variability of viruses and essential for evolution of most RNA viruses. In this study, based on RDP5 analysis, five potential recombinant events distributed non-randomly along the HoBiPeV genome were found only among the strains of HoBiPeV-a clade, which resulted in emergence of four recombinant viruses. Our findings here are inconsistent with a recent study, where the recombination events were found only among the strains of HoBiPeV-a clade [43]. The only difference was that six potential recombinant events were found in place of five found here as HoBiPeV strain Italy-1/10 (HQ231763) was found to be a triple recombinant in the previous study [43]. This may be due to the differences in analyses methods used in both studies. However, no recombination event was found among the analysed strains of HoBiPeV-c, d and e clades. Although uncommon, both heterologous and homologous recombination events (between different genotypes) have been reported for pestiviruses BVDV-1, BVDV-2 and CSFV under natural replication [50]. Hence, further studies may elucidate whether the same is also true for HoBiPeV.

## 5. Conclusions

In this study, we determined full genomic sequences of HoBiPeV strains of three novel and divergent clades (c, d and e) circulating in India and conducted the first full-genome-sequence-based genetic and evolutionary analyses of HoBiPeVs. Phylogenetic analyses confirmed the existence and independent evolution of four HoBiPeV main clades (a, c, d and e) globally, with inter-clade genetic divergence rates ranging between 13.0% and 18.2%. Our Bayesian molecular clock estimates revealed that HoBiPeV most likely originated in India, with a dated tMRCA of 1938 (1762–2000), suggesting a more recent origin of HoBiPeV. The rate of evolution of HoBiPeVs was estimated to be 2.133 × 10^−3^ subs/site/year for the full genome, whereas the evolution rate varied widely at the individual-gene level. Selection pressure analyses showed evidence of strong purifying (negative) selection, suggesting an important role of negative selection in HoBiPeV evolution for the first time. However, we also identified positively selected sites, mostly in *E2*. The findings of this study provide new insights into HoBiPeV origin and evolutionary history that may benefit in understanding host–pathogen interactions and developing newer therapeutics and vaccines for control of this emerging bovine pestivirus.

## Figures and Tables

**Table 1:** Summary of sequencing data of three complete HoBiPeV genomes (clade c, d and e) determined in this study and their comparison with globally circulating HoBiPeV strains in length of genome, polyprotein and individual genes.

S.No.	Strain	Acc. No.	Size (bp)														
			Polyprotein	5′-UTR	N^pro^	Capsid	E^rns^	E1	E2	P7	NS2-3	NS4A	NS4B	NS5A	NS5B	3′-UTR	Size of Genome
1	Ind/TN-1214/19	OQ411019	11,700	370	504	303	681	585	1119	210	3408	192	1041	1491	2166	189	12,259
2	IndABI15385/12	OQ411020	11,700	393	504	303	681	585	1119	210	3408	192	1041	1491	2166	279	12,372
3	IndBHA5309/12	OQ411021	11,700	373	504	303	681	585	1119	210	3408	192	1041	1491	2166	178	12,251
4	D32/00_HoBi	AB871953	11,700	367	504	303	681	585	1119	210	3408	192	1041	1491	2166	198	12,265
5	JS 12/01	JX469119	11,700	311	504	303	681	585	1119	210	3408	192	1041	1491	2166	134	12,145
6	Ch-KaHo/cont.	JX985409	11,700	380	504	303	681	585	1119	210	3408	192	1041	1491	2166	199	12,279
7	Th04_Khonkaen	FJ040215	11,700	383	504	303	681	585	1119	210	3408	192	1041	1491	2166	254	12,337
8	LVRI/cont	KC297709	11,700	383	504	303	681	585	1119	210	3408	192	1041	1491	2166	199	12,282
9	Italy-1/10-1	HQ231763	11,700	276	504	303	681	585	1119	210	3408	192	1041	1491	2166	128	12,104
10	PB22487	KY762287	11,700	256	504	303	681	585	1119	210	3408	192	1041	1491	2166	94	12,050
11	SV478/07	KY767958	11,700	370	504	303	681	585	1119	210	3408	192	1041	1491	2166	128	12,266
12	SV757/15	KY683847	11,700	380	504	303	681	585	1119	210	3408	192	1041	1491	2166	200	12,280
13	Italy 68/13 NCP	KJ627179	11,700	384	504	303	681	585	1119	210	3408	192	1041	1491	2166	159	12,243
14	Italy 83/10 ncp	JQ612704	11,700	384	504	303	681	585	1119	210	3408	192	1041	1491	2166	159	12,243
15	HN 1507	KU563155	12,006	391	504	303	681	585	1119	210	3714	192	1041	1491	2166	159	12,556
16	LV127-29/16MA	MH410815	11,700	346	504	303	681	585	1119	210	3408	192	1041	1491	2166	50	12,096
17	LV03/12	MH410816	11,700	277	504	303	681	585	1119	210	3408	192	1041	1491	2166	55	12,032
18	LV168-29/16RN	MH410813	11,700	334	504	303	681	585	1119	210	3408	192	1041	1491	2166	50	12,184
19	LV168-36/16RN	MH410812	11,700	391	504	303	681	585	1119	210	3408	192	1041	1491	2166	204	12,295
20	LV125-8/16MA	MH410814	11,700	254	504	303	681	585	1119	210	3408	192	1041	1491	2166	202	12,156
21	Italy-68/13cp	KJ627180	12,006	384	504	303	681	585	1119	210	3714	192	1041	1491	2166	159	12,549
22	Italy 83/10 cp	JQ612705	12,006	384	504	303	681	585	1119	210	3714	192	1041	1491	2166	159	12,549
23	Italy-129/07	KC788748	11,700	276	504	303	681	585	1119	210	3408	192	1041	1491	2166	142	12,118

**Table 2:** Unique amino acid changes observed in Indian HoBiPeV strains of clades c, d and e compared to the HoBiPeV-a strains analysed in this study.

Genes	HoBiPeV Strain/Clade (Amino Acid Positions)			
	IndABI15385/12 (Clade-c)	IndBHA5309/12 (Clade-d)	Ind/TN-1214/19 (Clade-e)	Common Changes Noticed in Analysed Strains of c, d and e
N^pro^	20, 36, 89, 107,150, 156	59, 62, 65,68, 70, 95, 127	32, 54, 75	Nil
C	20, 95, 100	6, 31, 92	94, 96	Nil
E^rns^	18, 41, 102, 105, 178, 179, 202	63	18, 41, 86, 134, 190	(I22T), (I122V), (E177D)
E1	91, 105, 107, 154, 181, 185	50, 72, 76, 79, 92, 93, 108	59,146, 162, 175	Nil
E2	23,140,186, 206, 380	39, 63, 68, 79, 207, 227, 231, 343, 376	32, 88, 179, 217, 220, 353	(D173S), (K265R)
P7	19, 24, 49, 61, 70	2, 10, 15, 18, 44, 54	39	(V42I), (S52N)
NS2	4, 49, 51, 73, 148, 55, 187, 210, 245, 280, 297, 299, 309, 334, 347, 369, 371, 412	10, 24, 115, 134, 136, 156, 265, 276, 338, 384, 385	94, 120, 262, 287, 313, 331, 337	(E217D), (S388T)
NS3	13, 99, 201	16, 72, 383,384	Nil	Nil
NS4A	63	2	9	Nil
NS4B	60, 92, 103	28, 43, 57, 63, 95, 157, 227	14, 35, 78	Nil
NS5A	109, 295, 304, 335, 358, 490	6, 20, 52, 53, 117, 123, 132, 150, 158, 190, 212, 256, 258, 270, 272, 282, 285, 295, 302, 320, 367, 429, 436, 460	113, 155, 281, 324, 334, 344, 421, 430, 471	(E313Q), (M416L), (G461E), (T462K)
NS5B	32, 236, 542, 642, 644	33, 60, 77, 84, 94, 99, 101, 105, 152, 153, 184, 241, 248, 278, 279, 357, 379, 381, 424, 597, 619	86, 104, 135, 187, 436, 580, 582, 655	(D14N), (I560V), (K661R), (Y675H), (L679I)

**Table 3:** Percent nucleotide and amino acid divergence of different coding regions of HoBi-like pestiviruses, including the strains of clades c, d and e analysed in this study.

Genomic Region	No. of Nucleotides	No. of Amino Acid	% nt Divergence	Mean nt Diversity	% aa Divergence	Mean aa Diversity
ORF	11,700	3899	0.10–18.8	7.1	0.30–11.6	4.6
N^pro^	504	168	0.00–21.2	7.4	0.00–15.7	5.4
C	303	101	0.00–19.9	6.4	0.00–8.1	2.8
E^rns^	681	227	0.00–17.2	6.5	0.00–10.1	4.2
E1	585	195	0.00–19.8	7.1	0.00–12.3	3.9
E2	1119	373	0.00–23.2	9.0	0.00–18.5	8.2
p7	210	70	0.00–24.3	7.2	0.00–25.7	6.0
NS2/NS3	3408	1136	0.10–17.6	6.7	0.30–10.2	3.6
NS4A	192	64	0.00–21.4	6.5	0.00–21.9	3.3
NS4B	1041	347	0.00–18.8	6.8	0.00–8.7	3.3
NS5A	1491	497	0.00–22.9	8.0	0.00–21.0	6.9
NS5B	2163	721	0.00–17.9	6.3	0.00–11.0	3.7

**Table 4:** HoBiPeV codon sites inferred to be under positive, episodic and negative selection pressure in this study, including the new sequences from HoBiPeV clades c, d and e. Positive selection pressure of codon sites was estimated by the SLAC (single-likelihood ancestor counting), FEL (fixed effects likelihood) and FUBAR (fast unbiased Bayesian approximation) methods. MEME (mixed effects model of evolution) was used to identify the codon sites under episodic diversifying selection. Criteria for strong evidence of selection: significance levels (*p* < 0.05).

Region	dN/dS	Sites under Pervasive Selection			Sites under Episodic Selection	% Sites under Purifying Selection
		SLAC	FUBAR	FEL	MEME	
ORF	0.126					21.8
N^pro^	0.167	114	114	114	61, 114	18.5
C	0.060				90	22.7
E^rns^	0.125				36, 38, 129, 139, 140	13.2
E1	0.105				120, 128, 132, 157, 194	22.1
E2	0.230	20	20, 89	20, 36, 38, 50, 89, 156, 179, 251, 255, 266, 283	19, 20, 21, 38, 89, 108, 156, 182, 251, 255, 266, 283, 286, 292, 304	21.2
p7	0.163					20.0
NS2/NS3	0.095			70, 1081	13, 29, 70, 105, 119, 132, 135, 158, 216, 271, 289, 294, 355, 462, 757, 907, 1044, 1077, 1081, 1123	23.2
NS4A	0.086				60, 62	21.9
NS4B	0.090			25, 400	11, 69, 242, 324	23.3
NS5A	0.158	304		304	274, 304, 324, 396, 397, 418, 473	22.1
NS5B	0.118	179, 203	203	179, 203	179, 203, 208, 461, 463, 465, 468	20.2

**Table 5:** Putative recombination events in HoBi-like pestiviruses, including the strains of clades c, d and e sequenced in this study using RDP5 software.

Recombinant	Major Parent	Minor Parent	Position in Alignment	Recombination Score	Detection Methods (*p* < 0.01)						
					RDP	GENECONV	BootScan	MaxChi	Chimaera	SiScan	3SEQ
MH410814	JX985409	Unknown	12293–12426	0.49	+	+	+	+	+	+	+
KC788748	HQ231763	KU563155	3808–5083	0.59	+	+	+	+	+	+	+
KU563155	JQ612705	Unknown	165–473	0.63	+	+	+	+	-	-	+
HQ231763	KC788748	Unknown	7465–8175	0.49	+	+	+	+	+	+	-
HQ231763	KY683847	Unknown	3818–4305	0.47	+	-	+	+	+	+	+
MH410813	AB871953	Unknown	2280–2379	0.51	+	+	+	+	+	-	+

N.B. No recombination events were evident for the strains of clades c, d and e (GenBank Acc. No. OQ411019, OQ411020 and OQ411021) sequenced in this study. + indicates evidence of recombination; - indicates no evidence of recombination.

**Table 6:** Estimates from Bayesian MCMC (Markov chain Monte Carlo) analysis for HoBi-like pestivirus genomes, including the genomes of clade c, d and e sequenced in this study. The evolutionary rates, tMRCA and RSPP values corresponding to the geographical locations are shown.

Genetic Region	Correlation	R Squared	Evolutionary Rate (95% HPD)	tMRCA (95% HPD)	Geographic Location	RSPP (Root State Posterior Probability)
Complete	0.4046	0.1637	2.133 × 10^−3^ (2.181 × 10^−4^–3.933 × 10^−3^)	1938 (1762–2000)	India	42.33
ORF	0.3997	0.1598	2.074 × 10^−3^ (2.833 × 10^−4^–3.759 × 10^−3^)	1942 (1800–1998)	India	40.86
N^pro^	0.4140	0.1714	2.389 × 10^−3^ (1.554 × 10^−4^–5.251 × 10^−3^)	1874 (1541–2000)	India	55.67
C	0.4331	0.1876	3.746 × 10^−3^ (6.922 × 10^−4^–8.008 × 10^−3^)	1983 (1950–2000)	Brazil	66.25
E^rns^	0.3713	0.1379	2.109 × 10^−3^ (4.242 × 10^−4^–4.043 × 10^−3^)	1963 (1889–2000)	Brazil	41.37
E1	0.4462	0.1991	3.279 × 10^−3^ (9.036 × 10^−4^–5.771 × 10^−3^)	1980 (1942–2000)	Brazil	57.16
E2	0.3817	0.1457	3.766 × 10^−4^ (4.377 × 10^−5^–7.034 × 10^−4^)	1211 (197–1994)	India	94.93
p7	0.3850	0.1483	3.95 × 10^−3^ (6.992 × 10^−4^–7.621 × 10^−3^)	1989 (1965–2000)	Brazil	68.30
NS2/NS3	0.4220	0.1780	2.632 × 10^−3^ (6.546 × 10^−4^–5.557 × 10^−3^)	1966 (1899–2000)	Brazil	42.05
NS4A	0.3156	0.0995	2.358 × 10^−3^ (6.946 × 10^−4^–4.001 × 10^−3^)	1987 (1965–2000)	Brazil	62.84
NS4B	0.3931	0.1545	2.503 × 10^−3^ (5.339 × 10^−4^–4.416 × 10^−3^)	1970 (1910–2000)	Brazil	64.23
NS5A	0.3833	0.1469	2.82 × 10^−3^ (6.695 × 10^−4^–4.969 × 10^−3^)	1975 (1927–2000)	Brazil	63.64
NS5B	0.3516	0.1236	2.277 × 10^−3^ (3.631 × 10^−4^–4.076 × 10^−3^)	1962 (1882–2000)	Brazil	50.98
5′-UTR	0.4479	0.2006	2.91 × 10^−3^ (1.029 × 10^−3^–4.974 × 10^−3^)	1983 (1954–2000)	Brazil	56.28

## Data Availability

All required data are available as texts and figures in main text of the article or in the Supplementary Materials. The sequence data generated in this study were submitted to GenBank and are available under Accession Numbers OQ411019–OQ411021.

## Supplementary Materials

The following supporting information can be downloaded at: https://www.mdpi.com/article/10.3390/v15030733/s1, Table S1: Full-genome sequences of HoBiPeV strains and other reference pestiviruses used in the dataset for genetic and evolutionary analyses in this study. Table S2: Nucleotide and amino acid sequence identity (%) of Indian HoBiPeV strains of clades c, d and e sequenced in this study with HoBiPeV-a strains and reference pestiviruses.
