# Supplementary material for: Whole-Genome-Sequence-Based Evolutionary Analyses of HoBi-like Pestiviruses Reveal Insights into Their Origin and Evolutionary History
Source: Viruses. 2023 Mar 11;15(3):733. doi: 10.3390/v15030733 (PMC10055830; doi:10.3390/v15030733)
Supplement: Supplementary file 1 [file viruses-15-00733-s001.zip › viruses-2266511-supplementary.pdf]

Supplementary Materials:

Supplementary Table S1. Full genome sequences of HoBiPeV strains and other reference pestiviruses used in the dataset for genetic and evolutionary analyses in this study.

Supplementary Table S2. Nucleotide and amino acid sequence identity (%) of Indian HoBiPeV strains of clades c, d and e sequenced in this study with HoBiPeV-a strains and reference pestiviruses.

Supplemetary Table S1. Full genome sequences of HoBiPeV strains and other reference pestiviruses used in the dataset for genetic and evolutionary analyses in this study.

**HoBiPeV**				
**S.No**	**Strain name**	**GenBank Acc. No**	**Country**	**Year**
1	Ind/TN-1214/19	**OQ411019**	India	2019
2	IndABI15385/12	**OQ411020**	India	2012
3	IndBHA5309/12	**OQ411021**	India	2012
4	D32/00_HoBi	AB871953	Brazil	2000
5	JS12/01	JX469119	China	2012
6	CH-KaHo/cont	JX985409	Switzerland	2005
7	Th04_KhonKaen	FJ040215	Thailand	2004
8	LVRI/cont-1	KC297709	South America	2012
9	Italy-1/10-1	HQ231763	Italy	2010
10	PB22487	KY762287	Brazil	2012
11	SV47807	KY767958	Brazil	2007
12	SV757/15	KY683847	Brazil	2015
13	Italy-6813ncp	KJ627179	Italy	2013
14	Italy-8310-ncp	JQ612704	Italy	2010
15	HN1507	KU563155	China	2015
16	LV127-29/16MA	MH410815	Brazil	2013
17	LV03/12	MH410816	Brazil	2011
18	LV168-29/16RN	MH410813	Brazil	2013
19	LV168-36/16RN	MH410812	Brazil	2013
20	LV125-8/16MA	MH410814	Brazil	2013
21	Italy-68/13cp	KJ627180	Italy	2013
22	Italy-83/10-cp	JQ612705	Italy	2010
23	Italy-129/07	KC788748	Italy	2007
**Other reference Pestiviruses**				
24	NADL (M31182); Osloss (M96687); Bega (KF896608); 890(U18059); NY 93(AF502399); Brescia (AF091661); Alfort 178 (X87939); BDV X818 (NC003679); BDV Giffhorn (KF925348); Pronghorn(NC024018); Bungowannah B869(NC023176); Giraffe H138 pestivirus(NC003678); Aydin-like(JX428945); NrPV/NYC-D23 (KJ950914)			

Supplementary Table S2. Nucleotide and amino acid sequence identity (%) of Indian HoBiPeV strains of clades c, d and e sequenced in this study with HoBiPeV-a strains and reference pestiviruses.

Genes			01. Ind BHA 5309	02. Ind ABI 15385	03. Ind_TN_1214_19	04. Th04_Khonkaen	05. D32 00 HoBi	06. JS12 01	07. HN 1507	08. CHKahocont	09. LVRI_Cont-1	10. Italy-1 10-1	11. PB22487	12. SV478 07	14. SV75715	15. Italy-6813ncp	16. Italy-68_13cp	17. Italy-8310-ncp	18. Italy-83_10-cp	19. LV03_12	20. LV125-8_16MA	21. LV127-29_16MA	22. LV168-29_16RN	23. LV168-36_16RN	24. NADL	25. 890	26. Brescia	27. X818	28. Giraffe	29. Pronghorn
Whole genome	1		***	81.2	82.3	82.1	81.9	82.0	81.7	82.1	81.6	81.3	81.9	81.8	82.0	81.6	84.0	81.7	81.7	81.8	81.1	81.8	81.6	82.0	68.8	68.8	69.0	69.1	68.6	63.4
	2		81.2	***	85.2	85.1	85.1	85.4	84.1	85.3	84.9	84.9	85.6	85.2	85.6	84.9	84.0	85.0	84.0	85.4	84.7	85.4	84.9	85.2	67.4	67.3	67.5	68.8	68.4	62.5
	3		82.3	85.2	***	87.2	87.2	87.0	86.9	87.1	86.8	86.6	87.0	86.9	87.2	86.9	86.9	87.0	86.9	86.9	86.4	86.9	86.9	87.2	68.8	69.1	68.8	68.9	68.9	63.2
5′-UTR	1	N	***	83	86.3	86.3	86.6	88.1	86.6	86.3	87.4	87.7	88.3	86.8	86	85.8	86.8	86.8	87.1	88.1	85.4	86.4	85.3	86.3	74	75.6	70.1	71.9	73.4	65.7
	2	N	83	***	87	88.6	86.9	90.4	85.7	86.1	87	90.9	92.2	87	87.8	85.9	86.4	86.4	86.7	90.6	90.2	91.6	87.1	86.4	70.8	71.9	69.8	68.9	72.9	69.7
	3	N	86.3	87	***	89.7	91.6	91.6	90	90.2	91.3	91.7	93.4	90.2	88	90.5	90.8	90.8	91.1	91.7	91.7	91.6	90.4	91.3	72.7	79.5	71.7	72.5	71.8	65.4
N^{pro}	1	N	***	79.8	81.2	79.2	79.6	80.6	79.6	80	79.6	79	80	79.8	79.6	79.6	79.6	79.8	79.4	80	80.2	79.8	79.2	79.2	66.3	64.1	67.5	67.1	62.9	63.3
		AA	***	84.5	85.7	85.7	86.9	85.7	87.5	86.9	83.9	86.3	86.3	86.3	86.3	88.1	87.5	87.5	87.5	87.5	86.9	85.7	86.3	86.3	64.9	64.9	70.2	66.1	62.5	56
	2	N	79.8	***	84.1	85.1	84.5	83.3	84.1	83.7	82.5	83.7	83.7	84.3	84.7	84.1	84.1	84.3	83.9	83.5	83.9	83.7	83.9	83.9	66.5	66.5	69.4	68.3	67.1	61.3
		AA	84.5	***	87.5	88.7	90.5	87.5	89.3	88.7	86.3	88.1	88.7	88.7	88.1	88.7	89.3	89.3	89.3	89.3	88.1	87.5	89.9	89.9	67.3	67.9	69.6	68.5	66.1	57.1
	3	N	81.2	84.1	***	86.9	86.9	86.5	86.1	85.5	85.3	85.5	85.9	85.3	86.5	86.1	86.1	86.3	85.9	86.3	85.5	84.9	86.7	86.7	65.3	66.7	66.9	67.9	66.3	65.1
		AA	85.7	87.5	***	91.7	93.5	91.1	92.3	91.1	88.7	91.1	91.7	91.1	91.1	91.7	92.3	92.3	92.3	92.9	91.7	90.5	92.9	92.9	66.7	67.3	69	67.9	64.9	57.7
Capsid	1	N	***	79.9	81.5	81.2	82.2	83.2	81.8	81.8	82.2	81.5	80.9	83.2	81.8	81.8	81.8	81.8	81.8	81.8	82.2	82.2	82.5	82.2	70.3	66.7	62.3	73.3	63.3	66.7
		AA	***	92.1	93.1	91.1	93.1	95	94.1	94.1	95	93.1	93.1	95	94.1	93.1	93.1	94.1	93.1	94.1	94.1	93.1	93.1	93.1	71.3	74.3	72.7	70	70.4	56.7
	2	N	79.9	***	84.8	82.5	85.1	85.8	87.1	85.8	86.1	86.8	83.8	85.5	85.8	86.5	86.5	87.1	86.5	85.8	86.1	85.8	85.1	85.5	62.7	68	68	72	60.9	60.5
		AA	92.1	***	93.1	90.1	93.1	95	94.1	94.1	95	93.1	92.1	95	94.1	93.1	93.1	94.1	93.1	94.1	94.1	93.1	93.1	93.1	70.3	74.3	72.7	68	73.5	59.8
	3	N	81.5	84.8	***	84.5	86.1	86.8	86.8	86.8	87.5	86.5	86.1	87.5	86.8	86.1	86.1	86.8	86.1	86.8	86.5	87.1	87.5	87.1	68	66.3	67	70	74.5	64.3
		AA	93.1	93.1	***	92.1	96	98	97	97	98	96	95	98	97	96	96	97	96	97	97	96	96	96	70.3	75.2	72.7	69	73.5	59.8
E^{rns}	1	N	***	84.7	85	84.3	84.4	83.4	84	84	84.1	83.6	85	83.6	84.8	83.7	83.7	84.1	83.7	82.8	83.3	84.6	84.4	84.4	71.5	71.5	71.2	70.2	72.5	62
		AA	***	95.6	96	95.6	93.8	91.6	94.7	94.7	93.4	93.4	95.2	93	94.6	93.8	93.8	94.7	93.8	93	93	94.7	94.3	94.3	78	79.7	75.8	80.2	81.1	58.6
	2	N	84.7	***	89.3	86.9	86.3	85.8	85.8	86.8	86	85	86.6	84.9	86.6	85.5	85.5	85.9	85.5	84.7	85.3	86.3	86.5	86.5	69.8	73.7	71.2	72.2	72.4	61.5
		AA	95.6	***	94.3	93.8	93	90.7	93.8	93.8	92.5	92.5	93.4	92.1	93.7	93	93	93.8	93	91.6	92.1	93	93.4	93.4	78	78.4	76.2	80.2	80.2	58.1
	3	N	85	89.3	***	90.3	88.4	88.1	88.7	89.3	89	88.3	89.3	88	89.4	88.4	88.4	88.8	88.4	86.9	87.7	88.3	88.4	88.4	70.3	72.5	70.8	73.3	70.9	61.7
		AA	96	94.3	***	93.4	92.1	89.9	93	93	91.6	91.6	93.4	91.2	92.8	92.1	92.1	93	92.1	91.2	91.2	93	92.5	92.5	77.5	79.3	73.6	79.7	81.9	58.6
E1	1	N	***	80.2	80.9	83.2	82.2	82.6	83.8	83.1	81.5	83.6	83.6	82.2	82.7	83.2	83.6	83.8	83.2	82.2	82.7	83.6	80.7	82.4	70.3	70.3	67.7	68.7	69.4	58.1
		AA	***	89.2	90.3	93.8	93.3	92.3	92.8	93.3	91.3	92.8	92.8	92.3	91.8	91.3	92.3	92.8	91.3	92.8	91.8	93.3	90.3	92.8	76.4	72.3	77.4	74.4	75.9	45.6

	2	N	80.2	***	83.1	85.1	84.6	84.4	85	84.3	84.4	85	84.6	83.4	84.3	84.4	84.8	85	84.4	83.6	83.9	83.8	81.4	83.2	68.9	69.6	68.2	70.3	68.7	56.9
		AA	89.2	***	90.3	92.3	90.8	90.8	91.3	91.8	90.8	91.3	91.3	90.8	90.3	89.7	90.8	91.3	89.7	91.3	90.3	90.8	87.7	90.3	75.9	73.8	75.4	77.4	74.4	48.2
	3	N	80.9	83.1	***	86	86.2	86.2	86.2	86	85.1	86.3	85.6	85.3	84.8	85.6	86.3	86.2	86	86	84.4	85.6	83.2	84.8	68.9	66.2	67.9	69.1	67.7	55.2
		AA	90.3	90.3	***	91.8	92.8	91.8	92.3	92.8	90.8	92.3	92.3	92.3	90.3	90.8	92.8	92.3	91.8	92.3	91.3	91.8	89.7	92.3	75.9	71.8	78.5	76.9	73.8	44.6
	1	N	***	80	78.9	78.4	76.9	78.6	77.5	77.1	77.9	77.7	77.7	77.7	77.7	77.7	78	77.7	78	77.2	77	78.6	77.7	77.7	62.6	61.6	58.7	61.2	60.3	48.1
		AA	***	85	84.7	83.9	82.8	83.1	82.3	82.8	83.1	83.1	84.2	82	83.9	83.1	83.4	83.1	83.4	83.4	81.8	82.8	84.5	84.5	63.5	58.3	59	57.9	58.7	43.8
	2	N	80	***	80.8	82.4	81.2	82.3	81.4	82.5	81.8	81.7	81.9	81.8	82.5	81.7	81.9	81.6	81.9	82.3	82	82.5	81.6	81.6	63.8	61.4	60.9	61.4	59.9	43.6
E2		AA	85	***	84.7	86.9	85	85.8	83.9	85	85.3	84.7	85.8	84.7	86.1	85	85	84.7	85	85.8	85	86.6	86.9	86.9	62.7	56.5	58.2	57.9	59.2	44.9
	3	N	78.9	80.8	***	84.1	84.2	84.3	84	84.5	84.6	84.3	83.6	84.3	84.6	84.2	84.4	84.2	84.4	84.5	84	84.5	85	84.8	62.4	60.8	58.9	51.9	61.4	42.9
		AA	84.7	84.7	***	88.2	88.5	87.4	86.3	87.9	88.5	86.9	88.5	89	89	87.1	86.9	86.6	86.9	88.7	87.4	87.9	90.3	90.3	61.9	58.3	58.4	58.2	59.5	43.8
	1	N	***	75.2	76.2	77.1	79.5	78.6	80.5	80	77.6	80.5	77.6	80	79.5	80	80.5	80.5	80.5	78.6	79	80.5	79.5	79.5	54.3	51	55.2	54.3	46.7	40
		AA	***	74.3	80	78.6	81.4	77.1	81.4	81.4	77.1	81.4	78.6	81.4	80	81.4	81.4	81.4	81.4	81.4	78.6	82.9	81.4	81.4	50	48.6	55.7	57.1	50	40
	2	N	75.2	***	80	80.5	81.9	80	82.4	81.4	80	82.4	81.9	80.5	82.4	81.9	82.4	82.4	82.4	81.4	82.9	81.4	81.9	81.9	59	59.5	59	57.6	60	41
P7		AA	74.3	***	84.3	81.4	85.7	81.4	85.7	85.7	81.4	85.7	87.1	85.7	84.3	85.7	85.7	85.7	85.7	82.9	82.9	84.3	85.7	85.7	54.3	54.3	64.3	65.7	54.3	41.4
	3	N	76.2	80	***	87.6	88.6	85.7	88.1	88.1	85.2	88.1	88.1	87.1	85.7	87.6	88.1	88.1	88.1	86.2	88.6	87.6	88.6	88.6	54.3	51	61	55.7	55.7	38.6
		AA	80	84.3	***	85.7	88.6	84.3	88.6	88.6	84.3	88.6	87.1	88.6	87.1	88.6	88.6	88.6	88.6	85.7	87.1	87.1	88.6	88.6	50	48.6	61.4	58.6	52.9	35.7
	1	N	***	80.6	85.4	87.4	86.8	86.5	87.6	77.5	86.6	81.9	87	87.6	86.9	85.9	85.7	86.1	86.1	86.5	85.9	86.8	85.9	87	51	53.6	56.7	57	51.5	46.6
		AA	***	77	81.8	81.9	82.1	81.8	78.2	81.9	81.2	80.7	82	82.9	82.6	82	72.7	82.2	72.8	82.5	82	81.8	81.6	82	52.1	60.7	59.4	64	51.4	54.5
	2	N	80.6	***	84.8	83.4	83.2	83.4	83.9	74.4	82.6	79.5	83.2	83.9	83.4	82.3	82.1	82.8	82.6	83.9	82.6	83.9	82.8	83.7	51.4	54.5	59.4	56.7	52.9	44.4
NS2		AA	77	***	79.6	79.7	79	78.8	70	79.5	78.4	77.6	78.8	78.9	79.5	79	69.8	79	70	79.5	79	78.8	78.4	78.9	53.7	61.4	60.9	60	53.3	42.6
	3	N	85.4	84.8	***	90.9	89.6	90.1	91.4	79.9	88.6	85.4	89.6	91.4	89.8	88.5	88.5	89.2	89	90.5	89.6	90.1	88.7	89.8	51.2	53.2	57.6	57.6	51.1	45.3
		AA	81.8	79.6	***	86.7	85.3	86.1	77.1	85.4	83.6	84.1	85.3	86	86.2	85.2	75.4	85.6	75.6	86.2	85.3	85.1	84.8	85.1	51.8	55	59.9	60.6	51.3	44.4
	1	N	***	86	88.5	88.4	89	89.3	88.7	88.8	88.8	88.1	88.7	88.7	88.5	88.4	88.6	88.6	88.5	88.5	88.3	88.8	88.4	88.4	77.6	75.7	77.6	77.3	77.8	70.4
		AA	***	98.5	98	97.2	98.1	98.1	97.8	97.8	97.8	96.6	98	98	98.2	97.7	97.7	97.8	97.5	98.1	97.4	98	98.1	98.1	91.2	89.9	90.6	92.2	92.1	78
	2	N	86	***	86.3	85.6	86.5	87	86.3	86.7	86.7	85.8	86.6	86.4	86.5	86.1	86.2	86.2	86.2	86.4	85.8	86.6	86.5	86.5	76.9	76.1	76.4	77.2	76.7	69.9
NS3		AA	98.5	***	98.4	97.8	98.7	99	98.5	98.5	98.5	97.4	98.5	98.7	99	98.4	98.4	98.5	98.2	98.8	98	98.7	98.8	98.8	91.7	89.9	90.6	92.2	92.5	78.5
	3	N	88.5	86.3	***	89	89	89.1	88.8	89	89	88.4	88.8	88.7	89	88.6	88.7	88.7	88.7	88.5	88.5	89.2	88.9	88.8	77	75.6	76.6	76.6	77.3	69.3
		AA	98	98.4	***	97.8	98.5	98.5	98.4	98.1	98.1	97.4	98.4	98.8	98.8	98.2	98.2	98.4	98.1	98.7	98.1	98.7	98.7	98.7	91.8	90.2	91.2	92.7	92.4	78.3
	1	N	***	83.3	83	84.4	82.6	81.8	82.5	81.8	81.4	81.2	82.1	82.1	82.4	82.6	82.4	82.3	82.4	83.2	83.3	83	83.2	83.4	69.9	71.2	72.1	71.5	72	66.8
		AA	***	91.9	92.8	93.4	93.9	93.7	94.2	94.2	92.8	91.4	94.8	93.1	93.9	94.2	94.2	93.6	93.9	94.8	94.8	93.9	94.2	94.5	77.8	77.5	78.7	79.3	79	68
	2	N	83.3	***	85.6	86.3	86.1	85.2	86.4	85.4	84.2	85.3	86	85.1	85.2	86.3	86.1	85.9	86	86.5	86	85.3	85.9	86.1	68.8	72.2	71.7	71.5	73.3	65.4
NS4B		AA	91.9	***	93.7	93.4	93.7	93.1	93.9	93.1	91.9	91.9	93.9	91.4	93	93.9	93.9	93	93.6	94.5	93.6	93.3	93.6	93.9	79	77.5	81.6	79.3	81	68.6
	3	N	83	85.6	***	88.1	86.9	86.5	87.3	87.1	86.6	86.3	86.7	85.5	87	87.4	87.2	87	87.1	87.1	87.2	87.3	86.9	87.1	70.1	72.1	70.8	70.5	72.9	59.1
		AA	92.8	93.7	***	94.5	94.8	94.8	95.4	94.8	93.3	93.1	95.4	92.5	94.8	95.4	95.4	94.8	95.1	96.2	95.4	95.1	95.1	95.4	77.5	77.2	80.1	79	79.8	68
	1	N	***	79.1	78.8	78.3	78.3	78.3	78.3	78.9	78.3	77.2	78.5	78.3	78.4	78.2	78.2	78.2	78.3	78.7	77.9	77.1	78.4	78.3	61.3	62.6	63.4	62.8	61	56.2
NS5A		AA	***	84.1	82.5	82.5	82.1	82.1	81.5	82.3	81.5	79.7	81.3	82.7	81.9	81.5	81.3	81.5	81.1	82.7	81.5	79.9	82.3	82.5	57.3	57.9	59.8	58.1	56.9	44.8

	2	N	79.1	***	82.9	83.1	85.3	84.8	84.7	84.7	84.2	83.4	85	84.5	85	84.6	84.6	84.5	84.6	84.2	84.7	84.1	84.9	85.2	61	63.2	62.8	62.3	61.4	56.8
		AA	84.1	***	88.5	88.9	88.3	87.7	87.1	87.9	86.7	84.1	87.7	87.3	88.1	87.1	86.9	86.7	86.7	87.9	87.7	86.3	88.1	88.3	56.5	57.3	59.6	57.3	55.1	45.3
	3	N	78.8	82.9	***	84.8	86	84.8	84.8	84.8	84.5	83.8	85.4	84.6	85	84.9	84.8	84.7	84.7	85	85.1	84.7	85.2	85.6	61.4	62.6	63.8	62.5	60.8	57
		AA	82.5	88.5	***	88.9	88.5	87.9	87.1	87.5	86.3	84.3	87.9	87.9	87.7	87.1	86.9	86.5	86.7	87.9	87.5	86.1	87.9	88.1	57.1	57.7	60.2	58.1	54.3	45.9
NS5B	1	N	***	83.2	83.3	82.9	83.1	83.1	82.5	82.7	82.5	82.5	82.9	82.4	83.1	82.5	82.5	82.4	82.5	82.7	82.5	82.3	82.9	83	69.6	70	70.3	70.1	69.5	64.3
		AA	***	90.3	91	89.8	90.3	91	90.7	88.9	90.4	90.7	90.4	90.4	90.9	90.7	91	91	91	90.4	90.4	90	90.7	90.8	74.4	74.2	76.2	74.5	75	64
	2	N	83.2	***	87.6	87.2	87.6	87.7	87.1	87.2	87.4	87.1	87.5	87.2	87.9	87.2	87.2	87.3	87.2	86.9	87.2	87.2	87.8	87.9	69.7	68.8	69.2	69.4	69.7	64.5
		AA	90.3	***	92.8	92.8	93.4	93.6	93.6	92.1	93.9	93.6	92.8	93.5	93.6	93.7	94	93.9	94	93.3	93.9	92.6	93.9	94.2	74.3	73.5	75.8	74.4	75.1	64.1
	3	N	83.3	87.6	***	88.1	88.3	88.6	88.5	88.3	88.2	88.5	88.5	88.5	88.9	88.5	88.5	88.3	88.5	88.1	88.7	88.1	88.4	88.3	70.3	70.3	69.6	69.7	70.8	65
		AA	91	92.8	***	92.8	93.3	93.9	93.6	92.2	92.8	93.6	92.9	93.6	93.8	93.3	93.6	93.5	93.6	93	93.2	92.8	93.3	93.5	74.9	74.3	76.2	75.2	76.2	64.5
